# Cusp-overlap view reduces conduction disturbances and permanent pacemaker implantation after transcatheter aortic valve replacement even with balloon-expandable and mechanically-expandable heart valves

**DOI:** 10.3389/fcvm.2023.1269833

**Published:** 2023-12-01

**Authors:** Tilman Stephan, Marvin Krohn-Grimberghe, Annika von Lindeiner genannt von Wildau, Christoph Buck, Michael Baumhardt, Johannes Mörike, Birgid Gonska, Wolfgang Rottbauer, Dominik Buckert

**Affiliations:** Department of Cardiology, Angiology, Pneumology and Internal Intensive Care, University of Ulm, Ulm, Germany

**Keywords:** aortic stenosis, conduction disturbance, cusp-overlap projection, implantation depth, implantation technique, permanent pacemaker implantation, TAVR, transcatheter aortic valve replacement

## Abstract

**Background:**

Conduction disturbances demanding permanent pacemaker implantation (PPI) remain a common complication after transcatheter aortic valve replacement (TAVR). Optimization of the implantation depth (ID) by introducing the cusp-overlap projection (COP) technique led to a reduced rate of PPI when self-expanding valves were used.

**Objectives:**

The aim of the present study was to determine if using the novel COP view is applicable for all types of TAVR prosthesis and results in a higher ID and reduced incidence of new conduction disturbances and PPI.

**Methods:**

In this prospective case-control study 586 consecutive patients undergoing TAVR with either balloon-expandable Edwards SAPIEN S3 (*n* = 280; 47.8%), or mechanically expandable Boston LOTUS Edge heart valve prostheses (*n* = 306; 52.2%) were included. ID as well as rates of periprocedural PPI and left bundle branch block (LBBB) were compared between the conventional three-cusp coplanar (TCC) projection and the COP view for implantation.

**Results:**

Of 586 patients, 282 (48.1%) underwent TAVR using COP, whereas in 304 patients (51.9%) the TCC view was applied. Using COP a significantly higher ID was achieved in Edwards SAPIEN S3 TAVR procedures (ID mean difference −1.0 mm, 95%−CI −1.9 to −0.1 mm; *P* = 0.029), whereas the final platform position did not differ significantly between both techniques when a Boston LOTUS Edge valve was used (ID mean difference −0.1 mm, 95%-CI −1.1 to +0.9 mm; *P* = 0.890). In Edwards SAPIEN S3 valves, higher ID was associated with a numerically lower post-procedural PPI incidence (4.9% vs. 7.3%; *P* = 0.464). Moreover, ID was significantly deeper in patients requiring PPI post TAVR compared to those without PPI [8.7 mm (6.8–10.6 mm) vs. 6.5 mm (6.1–7.0 mm); *P* = 0.005]. In Boston LOTUS Edge devices, COP view significantly decreased the incidence of LBBB post procedure (28.1% vs. 47.9%; *P* < 0.001), while PPI rates were similar in both groups (21.6% vs. 25.7%; *P* = 0.396).

**Conclusion:**

The present study demonstrates the safety, efficacy and reproducibility of the cusp-overlap view even in balloon-expandable and mechanically-expandable TAVR procedures. Application of COP leads to significantly less LBBB in repositionable Boston LOTUS Edge valves and a numerically lower PPI rate in Edwards SAPIEN S3 valves post TAVR compared to the standard TCC projection. The results should encourage to apply the COP view more widely in clinical practice.

## Introduction

**Figure 1:**
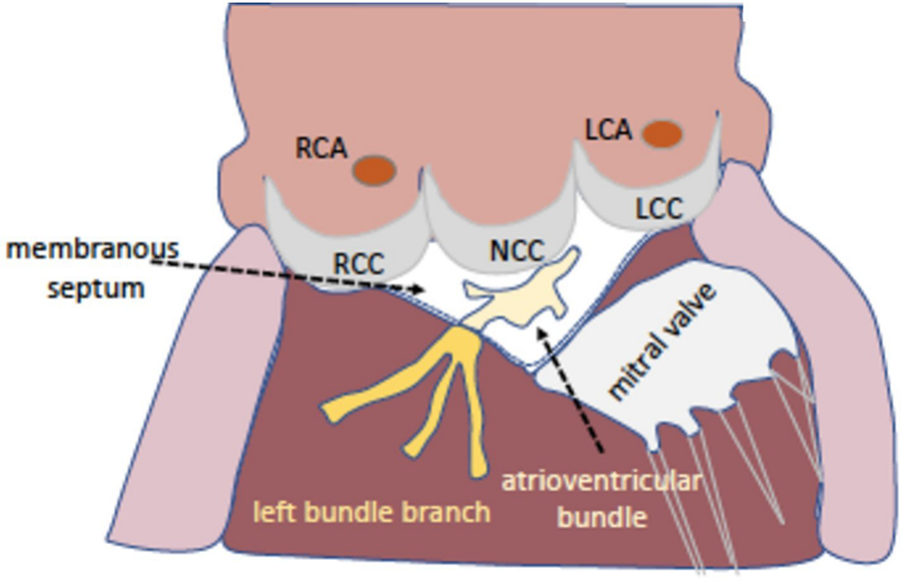
The aortic valve and the conduction system. The proximity of the aortic valve and the cardiac conduction system explains the occurrence of new conduction disturbances after transcatheter aortic valve replacement. Illustration showing the localization of the membranous septum and the left bundle branch between the right- and the non-coronary cusp. LCC/NCC/RCC, left-/non-/right-coronary cusp; LCA/RCA, left/right coronary artery.

Within the last two decades, transcatheter aortic valve replacement (TAVR) has revolutionized the management of symptomatic severe aortic stenosis (1–6). Continuous enhancements in all areas have allowed expansion from high-risk and inoperable patients to patients at all levels of surgical risk (7, 8). However, conduction disturbances demanding permanent pacemaker implantation (PPI) remains a common finding following TAVR with a reported incidence up to 36% before discharge (9–14). The main reason is the anchoring mechanism of most transcatheter heart valves (radial force) and the proximity of the implantation site to the cardiac conduction system resulting in high-grade atrioventricular block and new onset left bundle branch block (LBBB) (Figure 1) (15). Previous studies highlight that conduction abnormalities and new pacemaker requirement were linked to worse clinical outcomes including increased mortality and heart-failure rehospitalization (16–20). In view of the younger TAVR population in recent years, long-term consequences of pacing will become increasingly important (16).

Nowadays, several predictors associated with increased risk for post-procedural PPI are described in literature, however most of them are not modifiable (12, 13, 16, 21, 22). Optimization of the implantation depth (ID) by introducing the cusp-overlap projection (COP) technique might be a viable approach to lower the risk of interaction with the conduction system (23, 24). In TAVR using a self-expanding Evolut series prosthesis, the novel COP technique led to a significant higher prosthesis release and was associated with a reduced risk of PPI (25–27).

The aim of the present study was to determine if the rationale and practicalities of using the novel cusp-overlap view is applicable for all types of TAVR prosthesis and results in reduced incidence of new conduction disturbances and PPI.

## Methods

This prospective case-control study included consecutive patients undergoing transfemoral TAVR for aortic valve disease in our high-volume hospital heart center between March 2019 to December 2020. The decision for TAVR was made by the interdisciplinary heart team according to the 2017 European Society of Cardiology/European Association for Cardio-Thoracic Surgery Guidelines for the management of valvular heart disease (28).

**Figure 2:**
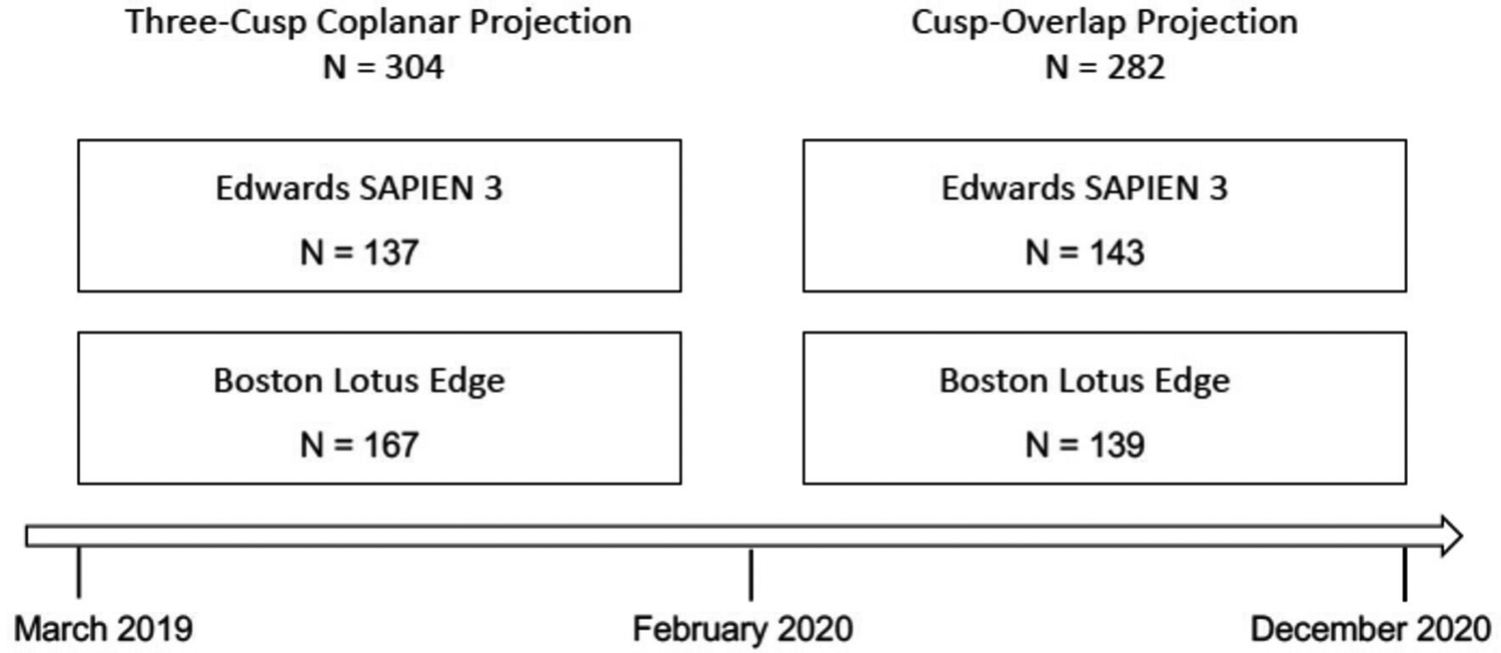
Study design. Between 03/2019 and 12/2020 consecutive patients without permanent pacemaker or implantable cardioverter defibrillator undergoing native valve transcatheter aortic valve replacement (TAVR) were included in the study. Prior to 02/2020 TAVR was performed in a three-cusp coplanar projection, and subsequently in a cusp-overlap projection.

TAVR was performed in a hybrid catheterization laboratory under conscious sedation by an experienced operator team of four interventionalists with a standardized procedure protocol according to current guidelines. The aortic valve prostheses were implanted under fluoroscopic guidance via the femoral access route. Patients received either a balloon-expandable Edwards SAPIEN 3 or a mechanically expandable Boston LOTUS Edge valve prosthesis. The decision for implanted device type was made by at least two experienced interventionalists and in accordance to current recommendations. Especially size (over-/undersizing), extent and morphology of calcification [particularly in the left ventricular out-flow tract (LVOT)] as well as access vessel situation were considered. There were no strict exclusion criteria for any of the prothesis types but the tendency to avoid balloon-expandable valves in case of severe LVOT-calcification (due to the risk of annulus rupture). Prostheses were sized using manufacturer recommendations, including annular and LVOT dimensions and location as well as severity of annular and LVOT calcification. Between March 2019 and January 2020 valve implantation was performed using the conventional three-cusp coplanar (TCC) view, whereas from February 2020 onwards the COP view was applied exclusively (Figure 2). The standard TCC view was reproduced based on computed tomography (CT) data. Thereafter, COP view was achieved by rotation around the center line. The proposed fluoroscopic angulations for optimal valve implantation were verified by angiography. The cusp-overlap view integrated a modified implantation technique according to the classic COP technique like wire management. For the sake of simplicity, only the term COP view will be used below.

Patients with pre-existing PPI as well as valve-in-valve procedures were excluded from this analysis.

The baseline characteristics and relevant periprocedural information of each patient were recorded. All patients received daily 12-lead electrocardiogram (ECG) to document serial changes in conduction as well as repeated laboratory testing and clinical examination. Transthoracic echocardiography was performed before and after the procedure to measure, amongst others, transvalvular aortic valve gradients and aortic regurgitation.

Furthermore, all patients underwent preprocedural ECG-gated 256 multislice contrast-enhanced CT, which was evaluated with a dedicated software (3mensio Structural Heart 9.1 software, Pie Medical Imaging B.V., Maastricht, The Netherlands). Besides the decision for the valve type and size, measurements of aortic annulus, LVOT, calcification and distance to coronary ostia, among others, were performed in accordance to the expert consensus document of the Society of Cardiovascular Computed Tomography (29). Moreover, the vascular access route was determined.

Primary clinical outcomes were new-onset of LBBB and PPI rates following TAVR as well as the measurement of ID comparing valve deployment with COP and TCC view. Furthermore, safety outcomes including device embolization, need for second valve implantation, coronary artery obstruction as well as moderate or severe aortic insufficiency post procedure were analyzed.

**Figure 3:**
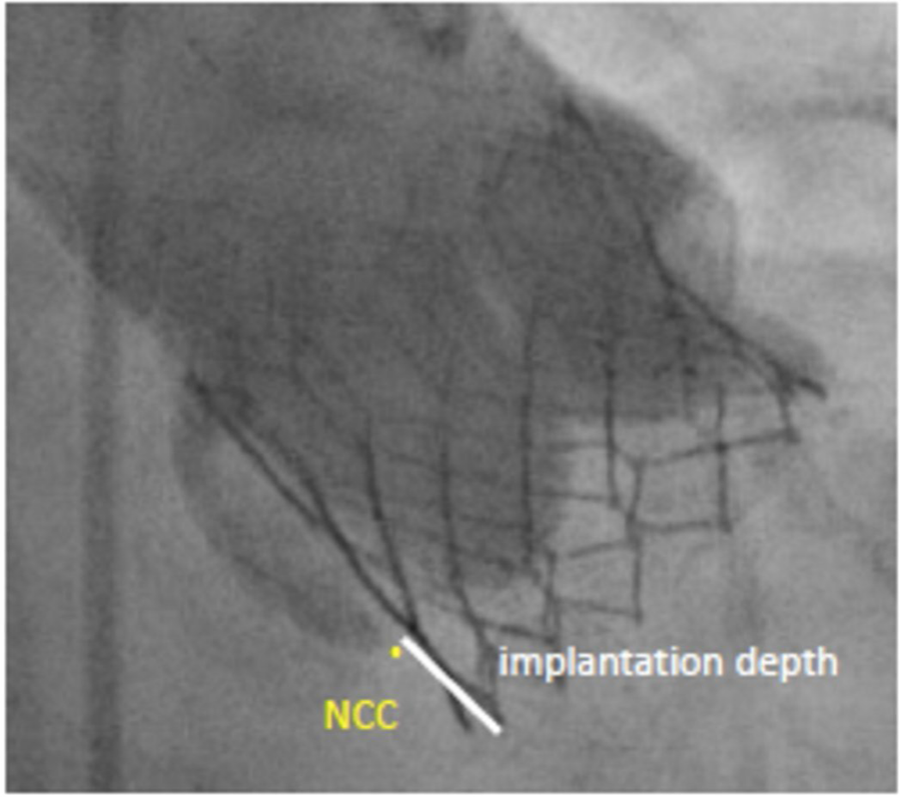
Implantation depth. Measurement from the non-coronary cusp (NCC) to the distal end of the intraventricular portion of the implanted valve.

PPI was considered in patients with persistent complete high grade atrioventricular block after TAVR. Assessment of the prothesis implantation was based on post-procedural evaluation of aortography and was carried out using a dedicated software (CAAS 7.4., Pie Medical Imaging, Maastricht, the Netherlands). ID was expressed as the maximal distance of the native aortic annulus plane on the side of the non-coronary cusp (NCC) to the most proximal edge of the implanted valve in the left ventricle (Figure 3). ID was measured in COP as well as TCC projection in all patients, with the greater distance used as the true ID.

All patients provided written informed consent to participate in the ULM TAVR-Registry. The study was approved by the local ethics committee and has been performed in accordance with the ethical standards laid down in the Declaration of Helsinki.

### Statistical analysis

Categorical data are presented as counts and percentages (%). Comparison of proportions was carried out using the *χ*^2^-test. Continuous variables are expressed as mean ± one standard deviation (SD). Continuous variables for two groups were compared with the Student's *t*-test. A *P*-value of <0.05 was considered to indicate statistical significance. Statistical analyses were performed using MedCalc software (MedCalc Version 20.210, MedCalc Software Ltd, Ostend; Belgium).

## Results

### Patient characteristics

**Figure 4:**
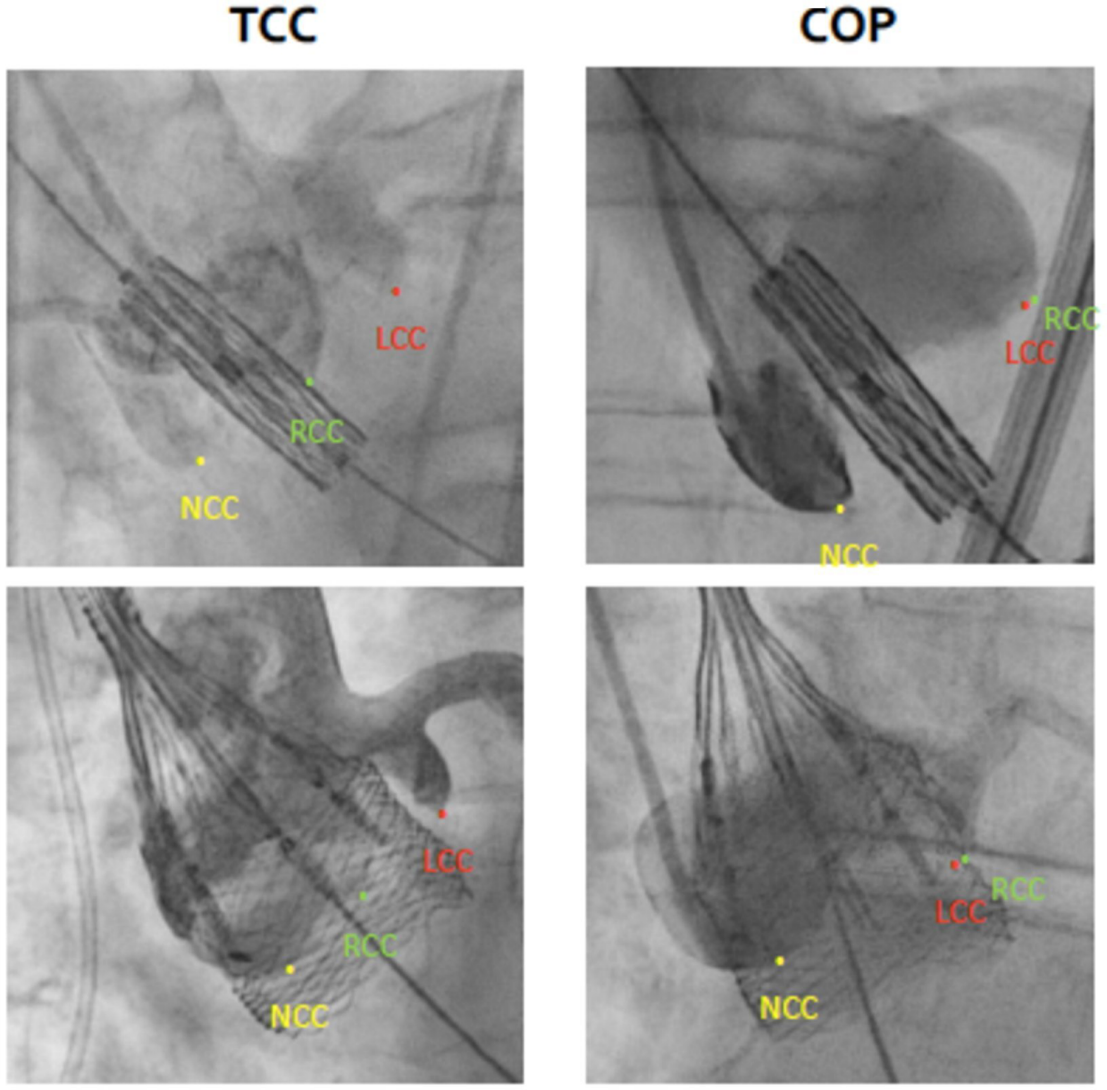
Anatomical distinctions between tricuspid-coplanar (TCC) and cusp overlap (COP) projections. Angiographic images of implantation in TCC (left) versus COP (right) for Edwards SAPIEN 3 (upper panel) and Boston Lotus Edge valves (lower panel). In the COP view the left- (LCC) and right-coronary cusp (RCC) overlap while the non-coronary cusp (NCC) is isolated on the left side.

A total of 586 consecutive patients with severe aortic stenosis undergoing TAVR via the femoral access were included in this study. Of those, 282 patients (48.1%) underwent TAVR using COP, whereas in 304 patients (51.9%) the TCC projection was applied (Figure 4). Baseline characteristics of both groups are displayed in Table 1. Median age was around 80 years [79.9 (79.0–80.7) years vs. 79.9 (79.2–80.6) years; *P* = 0.924] and sex ratio was well balanced (male 60.9% vs. 57.8%; *P* = 0.727). Mean STS-score was 3.3 [3.1–3.6] [3.3 (2.9–3.7) vs. 3.4 (3.1–3.7); *P* = 0.857] and 12.4%/13.2% of patients had LBBB/right bundle branch block (RBBB) prior to TAVR (13.4% vs. 11.5%; *P* = 0.608 and 13.8% vs. 12.6%; *P* = 0.709; respectively). Besides slight differences in mean and maximum transaortic pressure gradient [44.4 (41.4–47.5) mmHg vs. 37.3 (35.6–39.0) mmHg, *P* < 0.001 and 69.9 (67.1–72.6) mmHg vs. 62.8 (60.1–65.4) mmHg; *P* < 0.001, respectively] as well as in left ventricular ejection fraction [52.2 (50.9–53.4) % vs. 48.3 (46.7–49.8) % *P* < 0.001] all other baseline characteristics were similar distributed in both populations.

**Table 1:** Patient characteristics.

	TCC	COP	*P*-value
Total (*n*)	304	282	
Age (years)	79.9 (79.2–80.6)	79.9 (79.0–80.7)	0.924
Male	57.8%	60.9%	0.727
BMI (kg/m^2^)	27.3 (26.8–27.9)	27.0 (26.2–27.8)	0.610
STS-PROM	3.4 (3.1–3.7)	3.3 (2.9–3.7)	0.767
Diabetes mellitus	28.1%	29.1%	0.857
Chronic kidney disease	61.6%	49.0%	0.167
Arterial hypertension	93.4%	87.3%	0.698
Coronary artery disease	60.0%	55.9%	0.725
NYHA class III/IV	67.7%	66.5%	0.945
Left ventricular ejection fraction (%)	48.3 (46.7–49.8)	52.2 (50.9–53.4)	**0**.**0001**
AV max. PG (mmHg)	62.8 (60.1–65.4)	69.9 (67.1–72.6)	**0**.**0003**
AV mean (PG mmHg)	37.3 (35.6–39.0)	44.4 (41.4–47.5)	**0**.**0001**
Atrial fibrillation	22.4%	24.5%	0.704
LBBB	11.5%	13.4%	0.608
RBBB	12.6%	13.8%	0.709
Edwards SAPIEN 3	45.1%	50.7%	0.425
Boston LOTUS Edge	54.9%	49.3%	0.480

Data are presented as percentages, counts or mean ± SD. Significant *p* values are presented in bold.

AV mean/max PG, aortic valve mean/max pressure gradient; BMI, body mass index; COP, cusp-overlap projection; LBBB, left bundle branch block; NYHA, New York Heart Association; RBBB, right bundle branch block; STS-PROM, Society of Thoracic Surgeons—Predicted Risk of Mortality; TCC, three-cusp coplanar projection.

In 280 TAVR procedures (47.8%) a balloon-expandable Edwards SAPIEN S3 valve was implanted, while in 306 cases (52.2%) a mechanically expandable Boston LOTUS edge valve was used (Figure 4). Detailed baseline data of both valve subgroups are shown in Supplementary Table 1. Notably, in patients undergoing Edwards SAPIEN S3 TAVR procedure baseline incidence of LBBB was significantly more frequently in the COP-subgroup (10.9% vs. 16.4%, *P*-value < 0.0001).

### Procedural and clinical outcome

#### Edwards SAPIEN S3

**Figure 5:**
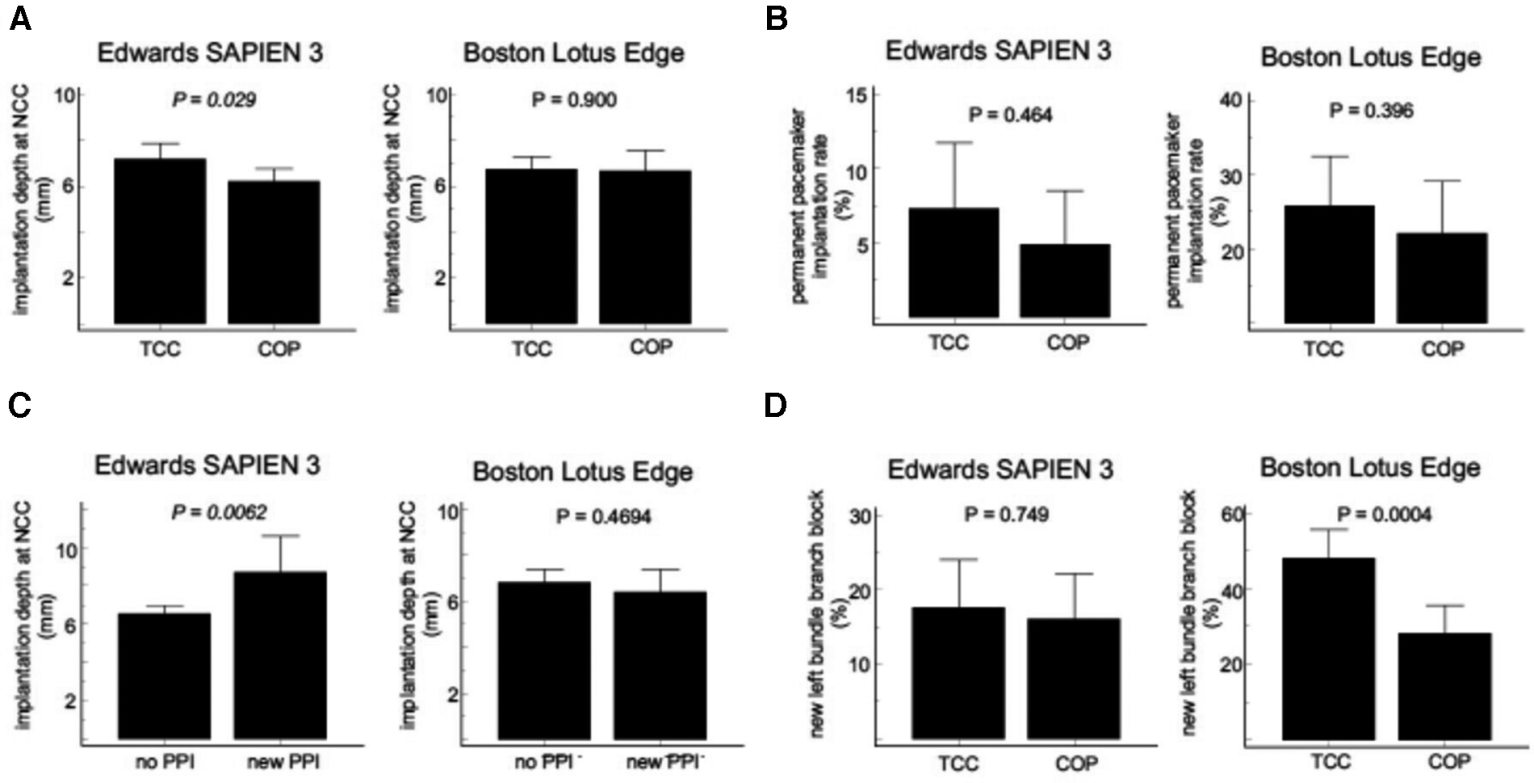
Comparison of cusp overlap projection (COP) and tricuspid coplanar projection (TCC) for TAVR with Edwards SAPIEN 3 and Boston LOTUS Edge valves. (**A**) implantation depth, (**B**) new permanent pacemaker implantation (PPI) rate, (**C**) implantation depth in patients with or without new PPI, (**D**) incidence of new-onset of left bundle branch block (LBBB).

In Edwards SAPIEN S3 TAVR procedures the final absolute mean ID was significantly smaller using COP view compared with the standard TCC projection (mean difference −1.0 mm, 95%-CI −1.9 to −0.1 mm; *P* = 0.029) (Figure 5A). The rate of new PPI following TAVR was numerically lower in the COP group than in the TCC group (7.3% vs. 4.9%; *P* = 0.464) (Figure 5B). Mean ID was significantly deeper in patients needing PPI post TAVR compared to those without PPI [8.69 mm (95%-CI 6.80–10.58 mm) vs. 6.52 mm (95%-CI 6.08–6.97 mm); *P* = 0.0052] (Figure 5C). Incidence of new-onset of LBBB post TAVR was similar distributed between both implantation techniques (TCC 17.5% vs. COP 16.1%, *P* = 0.752) (Figure 5D). Residual mean transaortic pressure gradient was also similar in both groups [TCC 12.0 mmHg (95%-CI 11.1–12.9 mmHg) vs. COP 12.7 mmHg (95%-CI 11.8–13.6 mmHg); *P* = 0.275]. Moreover, there were no device embolization, need for second valve implantation, coronary artery obstruction or moderate or severe aortic insufficiency post procedure in both cohorts (Table 2).

**Table 2:** Valve function and major complications.

	Edwards SAPIEN 3			Boston Lotus Edge		
	TCC	COP	*P*-value	TCC	COP	*P*-value
AV max. PG (mmHg)	22.3 (20.8–23.8)	23.4 (21.9–25.0)	0.303	23.5 (21.8–25.2)	25.0 (23.2–26.9)	0.213
AV mean PG (mmHg)	12.0 (11.1–12.9)	12.7 (11.8–13.6)	0.275	12.7 (11.7–13.6)	14.1 (13.0–15.0)	0.056
Aortic regurgitation grade II/III	0%	0%	n/a	0%	0%	n/a
Device embolization	0%	0%	n/a	0%	0%	n/a
Coronary obstruction	0%	0%	n/a	0%	0%	n/a

Data are presented as percentages or mean ± SD.

AV mean/max PG, aortic valve mean/max pressure gradient; COP, cusp-overlap projection; TCC, three-cusp coplanar projection.

#### Boston LOTUS edge

In patients receiving the Boston LOTUS Edge prothesis the final absolute mean ID did not differ significantly between both implantation views (mean difference −0.1 mm, 95%-CI −1.1 to +0.9 mm; *P* = 0.890) (Figure 5A). PPI rates following TAVR were 21.6% in the COP group and 25.7% in the TCC group (*P* = 0.396) (Figure 5B). Mean ID was also similar between patients with and without need for new PPI [6.38 mm (95% CI 5.38–7.39 mm) vs. 6.81 mm (95% CI 6.24–7.38 mm); *P* = 0.469] (Figure 5C). Post-procedure, the COP view was associated with a significant reduced incidence of LBBB (28.1% vs. 47.9%; *P* < 0.001) compared to the conventional TCC projection (Figure 5D). Residual mean transaortic pressure gradient was similar distributed in both groups [TCC 12.7 mmHg (95%-CI 11.7–13.6 mmHg) vs. COP 14.1 mmHg (95%-CI 13.0–15.0 mmHg); *P* = 0.056]. In both cohorts, device embolization, need for second valve implantation, coronary artery obstruction or moderate or severe aortic insufficiency did not occur (Table 2).

## Discussion

This is the first study analyzing the impact of the novel cusp-overlap view on conduction disturbances and PPI rates when performing TAVR with both balloon-expandable and mechanically expandable heart valves. The main findings can be summarized as follows: (1) Compared with the standard TCC projection, the COP view was associated with a significantly higher ID resulting in numerical less PPI after balloon-expandable TAVR. Moreover, ID was significantly higher in patients requiring PPI post TAVI compared to those without. (2) In mechanically expandable heart valves ID and rate of PPI were unaltered by using COP, however, less LVOT interference led to significantly reduced incidence of new onset of LBBB post procedure compared to TCC. (3) The periprocedural risk was low and similar distributed between both implantation techniques irrespective of the used valve type.

Nowadays, TAVR offers a safe and viable alternative to surgery for the management of severe and symptomatic AS at all levels of surgical risk (1–8). However, despite major enhancements, conduction disturbances demanding PPI remain a common and challenging complication after TAVR with the potential for long-term patient harm including increased early and late all-cause mortality as well as higher risk of heart failure rehospitalization (9–11, 13, 16–18). Especially since the therapy is increasingly moving toward patients with lower surgical risk and longer life expectancy, reduction of the persistently high PPI rates is one of the key issues in the field of TAVR (1, 2, 16).

To date, numerous risk factors for PPI after TAVR are well known, including amongst others LVOT calcification, preexisting conduction abnormalities like RBBB, left anterior hemiblock or first-degree atrioventricular block as well as the choice of valve prosthesis, degree of prothesis oversizing, short membranous septum length and depth of implantation of the new valve (12, 13, 16, 21, 22, 30). Besides the right prosthesis choice and oversizing ratio in some extent, especially the optimization of the ID represents a promising approach to reduce the risk of PPI after TAVR (23, 31). Due to the location of the conduction system with its bundle of His and the left bundle branch just below the annulus plane, a higher valve ID in the LVOT can reduce the risk of interaction with the conduction tissue and consequently the PPI risk after TAVR (15, 32, 33). Since just a few millimeters in ID can cause a large difference in PPI rates, precise imaging of the aortic root during valve implantation is an essential but challenging step for optimal valve positioning (10, 24).

The novel cusp-overlap approach, which was firstly reported at a conference in 2016 and systematically introduced in 2018, was suggested to enable better visualization and more accurate guiding of the optimal prosthesis ID during valve deployment (24, 32, 34, 35). In contrast to the standard TCC view, in which the right coronary cusp (RCC) is in the middle of the left (LCC) and the non-coronary cusp (NCC), the technique is based on overlapping the RCC and LCC, and thus isolating NCC (32). The anticipated advantages provided by the COP view are amongst others the elimination of the parallax of the delivery system, a better visualization of the NCC, achievement of a true coplanar view, elongation of the LVOT and consequently an accurate evaluation of an optimal and higher ID of the valve prosthesis (24, 32, 33, 36).

Due to promising results from previous studies the COP view has been widely extended in clinical practice, however data predominantly exists for the self-expanding valves (25–27). In a large meta-analysis with 3,647 patients from 11 studies undergoing self-expanding TAVR COP was associated with a significantly reduced PPI rate and higher ID compared to the standard TCC view (23). In the recently published Optimize PRO study with 400 patients receiving Evolut PRO/PRO + (Medtronic) self-expanding valves Grubb et al. displayed that the use of TAVR care pathway and COP resulted in favorable clinical outcomes with low PPI rates of only 9.8% at 30 days (37). The present study corroborates and further extends these findings.

We demonstrated that utilizing the COP view for valve implantation in TAVR with the balloon-expandable Edwards SAPIEN S3 prosthesis also resulted in a significantly higher ID compared to the standard TCC projection. Furthermore, higher valve implantation was associated with a favorable trend toward lower PPI rates, however without reaching statistical significance. To test if more patients might have allowed for a statistically significant difference, we compared the ID of patients receiving a PPI post TAVR with patients who did not. The latter group had a significantly lower ID, which is in line with data from Sammour et al. showing a similar reduction in the PPI rate of Edwards SAPIEN S3 valves with an alternative implantation technique to achieve higher valve implantation (RAO-CAU fluoroscopic projection) (38). Nevertheless, besides the COP view there are certainly several other factors affecting the implantation height like wire management or the learning curve every operator has to complete when adopting the technique. Moreover, prothesis specific behavior during implantation (tendency to migrate into ventricle vs. the aorta) may have led to different implantation heights.

In contrast to the Edwards SAPIEN S3 prosthesis, in TAVR using a mechanically-expandable Boston LOTUS Edge valve final ID did not differ between the new COP and the standard TCC view. The most likely reason for the unchanged ID is the distinct technique used for releasing the LOTUS valve during deployment. Boston LOTUS Edge valves were simultaneously released from their aortic and ventricular ends during mechanical expansion. As a result, it was more challenging to target and center the stretched-out valve. Since the valve tended to be dragged down as soon as the ventricular portion of the valve made contact with the LVOT, the final ID was less predictable compared to Edwards SAPIEN valves. Furthermore, parts of the LOTUS valve had to be positioned beneath the annulus to securely anchor the valve to avoid embolization as well as to reduce paravalvular aortic regurgitation. Due to its unique mechanical expansion during implantation as well as high radial force LOTUS Edge valves had a considerably higher rate of PPI compared to Medtronic Evolut and Edwards SAPIEN valves (1, 2, 39, 40). The observed PPI rate of 23.7% following Boston Lotus Edge TAVR in the present study agrees with data of previous trials (39, 41).

Remarkably, although higher ID for Boston Lotus Edge TAVR could not be achieved by using the COP view, postprocedural LBBB was significantly reduced from 47.9% to 28.1% compared to the TCC projection. Previous studies have also shown a lower incidence of new-onset of LBBB post TAVR using COP for deployment of a Medtronic Evolut Pro prosthesis, while the LBBB rate did not changed for Edwards SAPIEN S3 valves in our study (25–27, 42). In contrast to the Edwards SAPIEN S3 valve, Boston LOTUS Edge and Medtronic Evolut Pro valves can be repositioned before their final release if the interventionalist is dissatisfied with the final platform position. As mentioned above, the COP view allowed for better visualization of the annular plane leading to less LVOT manipulation during valve deployment and thus less trauma to the conduction system for self-expanding valves.

LBBB is a known marker of poorer long-term survival and leads to an intraventricular desynchrony potentially resulting in an impairment of the left ventricular function. In former studies new-onset of LBBB post TAVR was associated with an increased risk of PPI at 1-year, higher risk of heart failure rehospitalization as well as increased cardiac death and early and late all-cause mortality (19, 20, 43–45). We note that Boston Scientific has withdrawn the Lotus Edge Aortic Valve System in 2020 due to complexities associated with its delivery system during the implantation procedure. Nevertheless, our results can hypothesize as ´proof of concept` for the implementation of the COP technique for other TAVR valves, when the application leads to improved outcomes even in this valve with the highest PPI rates post procedure. Moreover, our study indicates that PPI rate also depends on other factors like the radial force.

However, the benefit of lower conduction disturbances and PPI post TAVR needs to be carefully weighed against possible adverse events, which may result from higher final valve ID (15, 24). Although not observed in this study, a higher implant theoretically possesses a potential increased risk for device embolization, paravalvular leakage, coronary artery obstruction as well as a complicated coronary reaccess. However, even in previous studies, the mentioned adverse events occurred very rarely after the application of COP and to a comparable extent as with the TCC view, which finally emphasize the safety and effectiveness of the COP view even in balloon-expandable and mechanically-expandable valves (25, 36, 38, 42).

### Limitations

The results of our study are to be interpreted with several confinements. First, this is a single-center observational study carrying all the inherent limitations ascribed to such type of design. Second, two non-contemporary cohorts were compared to evaluate differences between COP and TCC view. Furthermore, study cohorts were not matched on all baseline variables. Third, TAVR with the standard TCC projection was performed earlier than with the COP view. Therefore, PPI rates may have influenced by operatorś learning curve as well as the development of technique and devices over the years. Fourth, despite the prospective study design, the ID was measured retrospectively. Moreover, post TAVR ID was only measured angiographically and not by CT. Fifth, persistent LBBB and atrioventricular block after TAVR were assessed in ECGs until hospital discharge. Data on duration or resolution at 30 days or one year were not available. Sixth, the decision to implant a permanent pacemaker was ultimately at the discretion of the local heart team. However, except for class I indication the threshold for choosing a permanent pacemaker may differ among physicians and even institutions. Seventh, the Lotus Edge Aortic Valve System has been withdrawn from the market in 2020. Therefore, our implications have to be interpreted as hypothesis generating. Lastly, we observed no relevant safety endpoint in the present analysis for both implant techniques, possibly caused by a too small number of included patients. Further large-scale and multicenter studies are needed to further evaluate the safety and effectiveness of the COP and to confirm our results.

## Conclusion

The present study demonstrates the safety, efficacy and reproducibility of the cusp-overlap view even in balloon-expandable and mechanically-expandable TAVR procedures. Compared to the standard three-cusp coplanar view, application of cusp-overlap view leads to higher implantation depth in Edwards SAPIEN S3 TAVR resulting in numerically lower rate of permanent pacemaker implantation as well as to less LVOT interference and trauma in repositionable Boston LOTUS Edge valves coming across with lower incidences of new-onset of LBBB. The results should encourage to apply the cusp-overlap view more widely in clinical practice irrespective of the used valve type.

## Data Availability

The original contributions presented in the study are included in the article/**Supplementary Material**, further inquiries can be directed to the corresponding author.
