# Supplementary material for: Cusp-overlap view reduces conduction disturbances and permanent pacemaker implantation after transcatheter aortic valve replacement even with balloon-expandable and mechanically-expandable heart valves
Source: Front Cardiovasc Med. 2023 Dec 1;10:1269833. doi: 10.3389/fcvm.2023.1269833 (PMC10722163; doi:10.3389/fcvm.2023.1269833)
Supplement: Supplementary file 1 [file Table1.docx]

| **Supplementary Table 1. Patient characteristics by valve type.** | | | | | | | |
| --- | --- | --- | --- | --- | --- | --- | --- |
|  | **Edwards SAPIEN 3** | |  |  | **Boston Lotus Edge** | |  |
|  | **TCC** | **COP** | **P value** |  | **TCC** | **COP** | **P value** |
| Total (n) | 137 | 143 |  |  | 167 | 139 |  |
| Age (years) | 79.2 (78.1-80.2) | 79.5 (78.2-80.7) | 0.690 |  | 80.5 (79.6-81.4) | 80.2 (79.1-81.3) | 0.609 |
| Male | 67.6% | 62.3% | 0.472 |  | 49.7% | 59.4% | 0.081 |
| BMI (kg/m^2^) | 27.5 (26.7-28.4) | 27.9 (26.6-29.2) | 0.657 |  | 27.1 (26.4-27.8) | 26.5 (25.5-27.6) | 0.355 |
| STS-PROM | 3.5 | 3.8 | 0.588 |  | 3.1 | 3.0 | 0.402 |
| Diabetes mellitus | 28.7% | 25.0% | 0.567 |  | 27.5% | 32.6% | 0.379 |
| Chronic kidney disease | 59.3% | 44.4% | 0.040 |  | 63.5% | 53.3% | 0.101 |
| Arterial hypertension | 96.0% | 86.4% | 0.037 |  | 91.5% | 87.7% | 0.355 |
| Coronary artery disease | 61.8% | 54.5% | 0.143 |  | 54.2% | 56.1% | 0.788 |
| NYHA class III/IV | 69.1% | 66.7% | 0.781 |  | 66.5% | 66.9% | 1.0 |
| Left ventricular ejection fraction (%) | 48.3 (46.0-50.5) | 50.2 (48.0-52.4) | 0.226 |  | 50.0 (48.3-51.8) | 52.6 50.9-54.4) | 0.043 |
| AV max. PG (mmHg) | 61.9 (58.3-65.5) | 67.7 (63.4-72.0) | 0.040 |  | 66.3 (62.6-69.9) | 70.8 (66.7-74.8) | 0.101 |
| AV mean PG (mmHg) | 36.6 (34.2-39.0) | 42.4 (39.4-45.3) | 0.003 |  | 39.6 (37.4-41.8) | 43.7 (40.8-46.6) | 0.022 |
| Atrial fibrillation | 26.5% | 27.3% | 0.851 |  | 19.2% | 21.7% | 0.994 |
| LBBB | 10.9% | 16.2% | <0.0001 |  | 12.0% | 10.5% | 0.650 |
| RBBB | 17.2% | 15.4% | 0.722 |  | 8.9% | 12.2% | 0.332 |
| Data are presented as percentages, counts or mean ± SD. Significant p values are presented in bold.  AV mean/max PG, aortic valve mean/max pressure gradient; BMI, body mass index; COP, cusp-overlap projection; LBBB, left bundle branch block; NYHA, New York Heart Association; RBBB, right bundle branch block; STS-PROM, Society of Thoracic Surgeons - Predicted Risk of Mortality; TCC, three-cusp coplanar projection. | | | | | | | |
